# Cold-Active, Heterotrophic Bacteria from the Highly Oligotrophic Waters of Lake Vanda, Antarctica

**DOI:** 10.3390/microorganisms3030391

**Published:** 2015-07-24

**Authors:** Nicole A. Vander Schaaf, Anna M. G. Cunningham, Brandon P. Cluff, CodyJo K. Kraemer, Chelsea L. Reeves, Carli J. Riester, Lauren K. Slater, Michael T. Madigan, W. Matthew Sattley

**Affiliations:** 1 Division of Natural Sciences, Indiana Wesleyan University, 4201 South Washington St., Marion, IN 46953-4974, USA; E-Mails: Nicole.VanderSchaaf@vai.org (N.A.V.S.); annamgcunningham@hotmail.com (A.M.G.C.); chelsealreeves@gmail.com (C.L.R.); carlijriester@trevecca.edu (C.J.R.); laurenkayslater@gmail.com (L.K.S.); 2 Department of Biology, MidAmerica Nazarene University, 2030 E. College Way, Olathe, KS 66062, USA; E-Mails: brandonpcluff@gmail.com (B.P.C.); ckraemer@email.arizona.edu (C.K.K.); 3 Department of Microbiology, Southern Illinois University, 1125 Lincoln Dr., Carbondale, IL 62901, USA; E-Mail: madigan@micro.siu.edu

**Keywords:** Antarctica, Lake Vanda, McMurdo Dry Valleys, psychrotolerant, halotolerant

## Abstract

The permanently ice-covered lakes of the McMurdo Dry Valleys, Antarctica are distinctive ecosystems that consist strictly of microbial communities. In this study, water samples were collected from Lake Vanda, a stratified Dry Valley lake whose upper waters (from just below the ice cover to nearly 60 m) are highly oligotrophic, and used to establish enrichment cultures. Six strains of psychrotolerant, heterotrophic bacteria were isolated from lake water samples from a depth of 50 or 55 m. Phylogenetic analyses showed the Lake Vanda strains to be species of *Nocardiaceae*, *Caulobacteraceae*, *Sphingomonadaceae*, and *Bradyrhizobiaceae*. All Lake Vanda strains grew at temperatures near or below 0 °C, but optimal growth occurred from 18 to 24 °C. Some strains showed significant halotolerance, but no strains required NaCl for growth. The isolates described herein include cold-active species not previously reported from Dry Valley lakes, and their physiological and phylogenetic characterization broadens our understanding of these limnologically unique lakes.

## 1. Introduction

Lake Vanda is a stratified lake about 75 m deep located in Wright Valley of the McMurdo Dry Valleys, Antarctica, a region that experiences subzero average temperatures and extremely low (~10 mm) annual precipitation [1]. As in other Dry Valleys lakes, metazoans and other more complex eukaryotic organisms are absent from Lake Vanda, and therefore biological processes in the lake are driven entirely by the activities of microorganisms. In addition, microorganisms inhabiting the lake are isolated from their surroundings by a permanent ice cover of approximately 3.5 m, which limits the penetration of light and inhibits wind mixing of the water column. As a result, the meromictic water column exhibits distinct vertical clines with defined zones of primary productivity [2].

**Figure 1:**
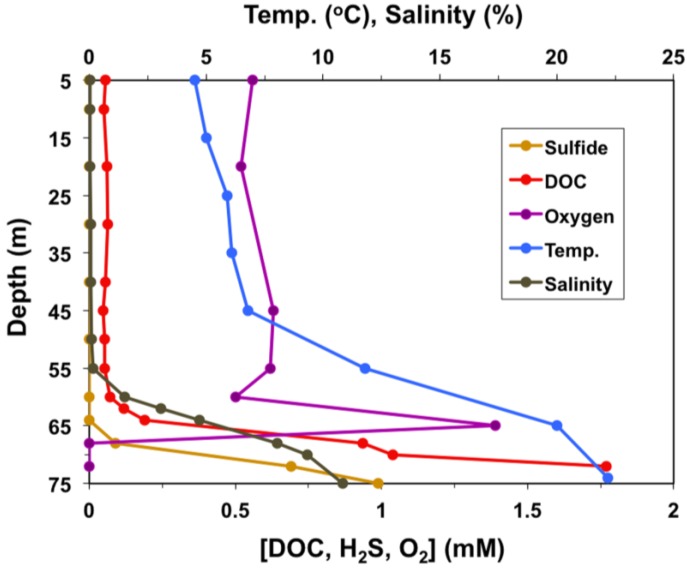
Physiochemical profile of the Lake Vanda water column. Due to the permanent ice cover on Lake Vanda, measurements begin at a depth of 5 m.

The waters of Lake Vanda range from fresh and cold (~4 °C) just below the ice cover to hypersaline and surprisingly warm (~24 °C) just above the sediments (Figure 1). At depths of 50–55 m, Lake Vanda waters are approximately 10–12 °C with a pH of 6.5–7 [3]. Levels of dissolved organic carbon (DOC) in Lake Vanda are quite low (<0.2 mg·C·L^−1^) down to about 60 m, and salinity is nearly undetectable to a depth of 55 m [4,5] (Figure 1). In addition, the lake is supersaturated with dissolved O_2_ to a depth of about 65 m [3], beyond which salinity, temperature, and sulfide increase to the sediments (Figure 1).

Compared to temperate aquatic ecosystems, our knowledge of microbial life in the permanently cold Dry Valleys lakes of Antarctica remains limited. Limnological and physicochemical properties of these unusual lakes have been well studied and have linked biogeochemical nutrient cycling to the activities and distributions of various microorganisms [2,3,6,7,8,9,10,11,12,13,14,15]. Although several studies have focused on the enrichment and isolation of bacteria from lakes in the Dry Valleys [16,17,18,19,20,21,22], few descriptions of bacterial isolates from Lake Vanda exist. Decades-old studies on the microbial communities of Lake Vanda reported the successful culture of some bacteria and even yeasts from Lake Vanda [23,24,25,26]; however, phylogenetic analyses were not included in these descriptions. More recently, Bratina *et al.* [27] described cultures of manganese-reducing bacteria, mostly of the genus *Carnobacterium*, from oxic portions of the Lake Vanda water column, and Tregoning *et al.* [28] described CaCl_2_-tolerant, halophilic bacteria isolated from the comparatively warm Lake Vanda brines of 60–72 m. In the latter study, the 15 mesophilic strains obtained comprised a single species most closely related to the aerobic marine γ-proteobacterium *Halomonas zincidurans*.

We have employed culture-based methods to obtain new isolates of heterotrophic bacteria from the cool and oligotrophic upper waters of Lake Vanda. Our analyses reveal species not previously recognized from any Dry Valley lake (including Lake Vanda) and compare these cold-active strains to previously described Dry Valley lake species. The halotolerant and psychrotolerant chemoorganotrophic bacteria described here grew well under the physiochemical conditions of their habitat and, therefore, likely contribute to DOC degradation and nutrient cycling in the water column of Lake Vanda.

## 2. Materials and Methods

### 2.1. Sample Collection and Analysis, Enrichment, and Isolation

Lake Vanda water samples were obtained at coordinates 77°31.685′ S/161°35.425′ E by using a gasoline-powered drill head fitted with joined sections of a 10-inch (25.4 cm) steel auger to create the sampling hole in the ~3.5-m lake ice cover. Water samples were collected in a 5 L Niskin sampling bottle and transferred to completely filled PVC or polycarbonate Nalgene™ bottles that had been previously washed with a 10% (v/v) HCl solution and thoroughly rinsed with distilled water. All samples were kept cold (~4 °C) but unfrozen in darkness from the time of their collection. Water column sulfide concentrations were determined as previously described [11] using the methylene blue colorimetric method of Trüper and Schlegel [29]. Additional physiochemical properties of the Lake Vanda water column were obtained from the National Science Foundation—funded McMurdo Long-Term Ecological Research Program online database [30].

Primary enrichment cultures were established by aseptically spreading 0.1 mL Lake Vanda water from a depth of 50 or 55 m onto petri dishes containing R2A agar (pH 7.2) [31] or Bacto Plate Count Agar [32]. Incubations took place in darkness at 4 °C, 8 °C, or room temperature (~22 °C). Colonies selected for isolation were purified by at least three successive platings. Isolated strains were transferred to and maintained in loosely screw-capped 10-mL tubes containing 3 mL of either a defined ammonium glucose medium (described below) or liquid medium R2A and incubated at 4–18 °C. To ensure sufficient O_2_ in cell suspensions, liquid cultures of isolated strains were mixed several times per week.

The ammonium glucose medium contained the following per liter of double-distilled water: glucose, 10 g; NH_4_Cl, 1 g; KH_2_PO_4_, 0.5 g; KCl, 0.2 g; MgSO_4_·7H_2_O, 0.2 g; CaCl_2_·2H_2_O, 0.05 g; and yeast extract, 0.01 g. All chemicals were reagent grade and obtained from Sigma-Aldrich, Co., St. Louis, MO or J.T. Baker Chemical Co., Phillipsburg, NJ, USA.

### 2.2. Morphology

Morphological determinations were made using a Leica Microsystems Model DM1000 phase-contrast light microscope, and high-resolution imaging was done on an FEI NOVA nanoSEM field emission scanning electron microscope (FEI Company, Hillsboro, OR, USA). Capsule stains were performed using the method of Maneval [33]. Motility was determined by microscopic observations and from assessing stab inoculations into semi-solid (0.35% agar) sulfide-indole-motility (SIM) medium (Becton, Dickinson and Company, Sparks, MD, USA) [32].

### 2.3. Physiological Studies

To determine the cardinal growth temperatures for isolated strains, triplicate 10-mL tubes of liquid medium R2A were inoculated and incubated for seven days at temperatures ranging from −2 °C to 37 °C; growth was assessed turbidimetrically. Growth rates were measured by periodic turbidity readings of cultures grown in the same way. Salinity tolerance was determined using liquid R2A media containing various levels of NaCl; cultures were incubated at 15 °C and visually inspected for turbidity for up to one month.

The ammonium glucose medium described above was used to test the utilization of various carbon sources. In these experiments, glucose was substituted with a variety of single carbon sources or yeast extract. Inoculated tubes were incubated at 15 °C for 10 days before scoring for growth. The potential for anaerobic growth was tested in all strains using anoxic media containing either dimethyl sulfoxide, sodium nitrate, or sodium fumarate (each at a final concentration of 10 mM) as an alternative electron acceptor. To achieve anoxic conditions, 1.2 mM (final) sodium sulfide was added to R2A media containing resazurin as redox indicator before being dispensed into completely filled screw-cap tubes, inoculated, and incubated at 15 °C for one month.

The potential for phototrophic growth of strain VP55 was tested in completely filled 150-mL bottles containing the following medium (per liter of double-distilled water): ethylenediaminetetraacetic acid (EDTA), 10 mg; MgSO_4_·7H_2_O, 0.2 g; CaCl_2_·2H_2_O, 0.075 g; NH_4_Cl, 0.75 g; NaCl, 1 g; Na_2_S_2_O_3_·5H_2_O, 0.1 g; sodium succinate, 2.0 g; sodium pyruvate, 0.5 g; sodium acetate, 0.5 g; yeast extract, 0.5 g; vitamin B_12_, 20 μg; trace elements [34], 1 mL; and K_2_HPO_4_, 1 g. Cultures were incubated for six weeks at 15 °C at four different incandescent light intensities before being scored for growth.

Starch hydrolysis was tested in complex media containing 1% (wt/vol) soluble starch [32]. SIM medium was used to test for indole formation by tryptophanase and also for sulfide production via cysteine desulfurase and/or thiosulfate reductase [32]. The possibility of gelatinase and urease synthesis was tested in complex nutrient media containing gelatin (40 g/L) or urea (20 g/L), respectively [32].

### 2.4. Phylogenetic Analyses

Genomic DNA was isolated from mid-log-phase cells, and small-subunit (16S) rRNA gene sequences were PCR amplified as previously described [16]. Controls containing no DNA were included in all PCR reactions, and 16S rRNA gene amplification products were confirmed to be of proper size (~1500 bp) by comparison to a 1 Kb DNA Ladder (Invitrogen, Carlsbad, CA, USA). Bacterial DNA from each strain was purified from gels using the QIAquick Gel Extraction Kit (QIAGEN Sciences, Germantown, MD, USA) according to the manufacturer’s instructions.

Sequence fragments of purified 16S rRNA genes were assembled, and multiple alignments were generated using UGENE version 1.11.0 [35], MUSCLE [36], and MEGA version 5.05 [37]. Positions with gaps or missing data were eliminated from each analysis. Phylogenetic relationships were determined using BLAST [38], sequence analysis functions of the Ribosomal Database Project [39], and the List of Prokaryotic names with Standing in Nomenclature [40]. MEGA5 was used to create maximum-likelihood phylogenetic trees with 1000 bootstrap replications using the Tamura-Nei distance correction model and the Nearest-Neighbor-Interchange (NNI) heuristic method [41].

Small-subunit rRNA gene sequences from the six strains of Lake Vanda bacteria isolated in this study were deposited into GenBank under the accession numbers shown in the phylogenetic tree figures. Cultures of the Lake Vanda isolates were preserved at −80 °C in growth medium containing 10% (final) dimethyl sulfoxide (DMSO) and are available from the corresponding author upon written request.

## 3. Results

### 3.1. Isolation and Morphology of Lake Vanda Strains

After a two-week incubation at temperatures ranging from 4 °C to room temperature, both pigmented and unpigmented colonies developed on plates of enrichment media inoculated with Lake Vanda water from depths of 50 or 55 m. Six strains that grew well were isolated in pure culture from these colonies and selected for further study.

**Figure 2:**
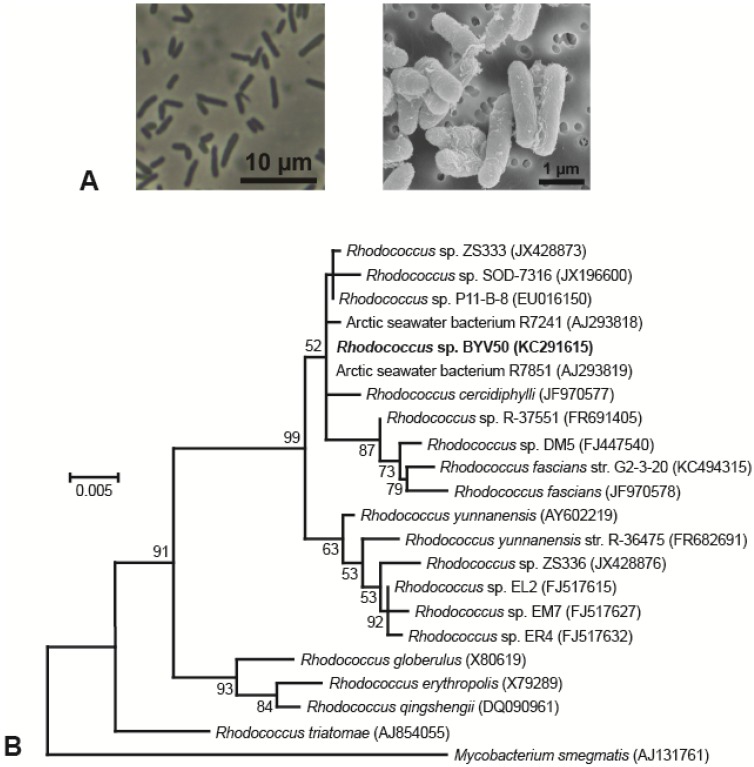
Morphology and phylogeny of *Rhodococcus* strain BYV50. (**A**) Phase-contrast micrograph (left) and scanning electron micrograph (right) showing cell morphology and arrangement; (**B**) Phylogenetic tree generated from 1435 nucleotide positions of the 16S rRNA gene. *Mycobacterium smegmatis* is the outgroup organism, and bootstrap values >50% are shown at the branching points. GenBank accession numbers are listed in parentheses.

**Figure 3:**
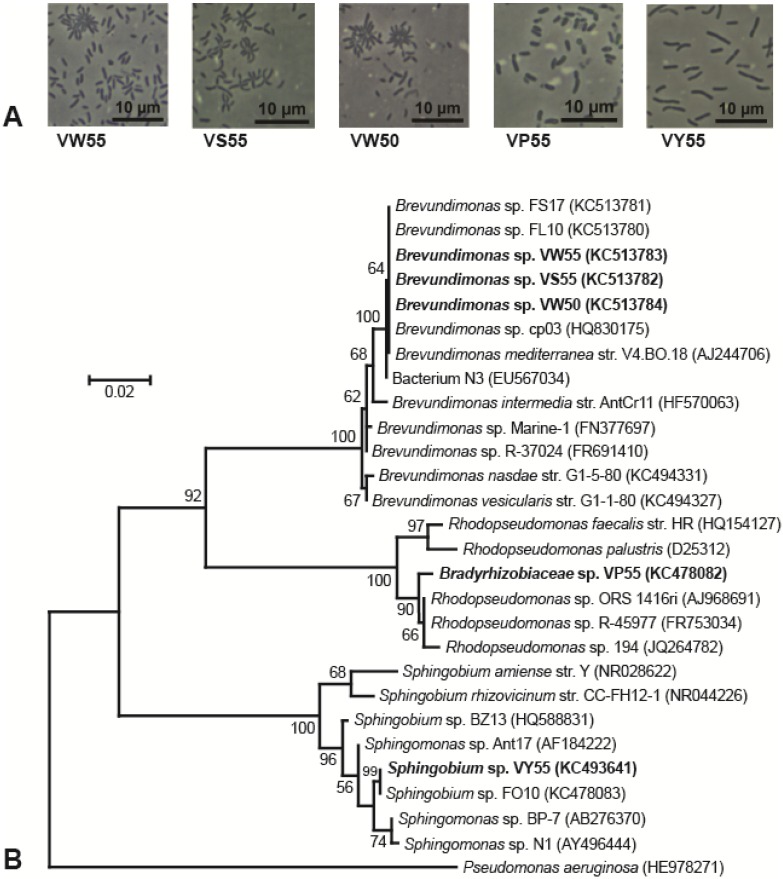
Morphology and phylogeny of gram-negative Lake Vanda isolates. (**A**) Phase-contrast photomicrographs showing cell morphology and arrangement of each strain; (**B**) Phylogenetic tree generated from 1369 nucleotide positions of the 16S rRNA gene. *Pseudomonas aeruginosa* is the outgroup organism, and bootstrap values > 50% are shown at the branching points. GenBank accession numbers are listed in parentheses.

When grown on plates of medium R2A, colonies of strain BYV50 appeared bright yellow to orange, strain VP55 was pink, strain VY55 was bright yellow, and strains VS55, VW50, and VW55 were not pigmented (Table 1). Colonies of five of the strains had entire margins, convex elevation, a smooth surface, opaqueness, and circularity; by contrast, colonies of strain VP55 were translucent and punctiform. Strains BYV50 and VP55 were nonmotile rods, whereas cells of strain VY55 were highly motile rods that appeared as single cells, short chains, or occasionally as rosettes of several cells tethered together. Cells of strains VW50, VW55, and VS55 were highly motile, short rods that were often arranged in rosettes of 8–15 cells. The major phenotypic properties of the Lake Vanda strains are shown in Table 1, and photomicrographs of all strains are shown in Figure 2A and Figure 3 A.

**Table 1:** Summary of major properties of Lake Vanda bacteria isolated in this study ^a^.

Property	*Rhodococcus* sp. BYV50	*Brevundimonas* spp. VW50, VW55, and VS55	*Bradyrhizobiaceae* sp. VP55	*Sphingobium* sp. VY55
Cell shape	Straight rod	Straight-to-curved rod	Straight rod, often tapered	Straight rod
Motility	−	+	−	+
Cell size (l × w, μm)	2–5 × 1–1.2	1.4–3.2 × 0.7–0.8	1.5–3.5 × 0.9–1.1	1.8–8 × 0.9–1
Gram stain reaction	+	−	−	−
Pigment	Bright yellow/Orange	None	Pink	Bright yellow
Temp. range (°C)	−2–32	−2–35	2–29	0–30
Temp. optimum (°C)	18–24	20	20	20
Salinity range (% NaCl)	0–10	0–6	0–1	0–6

^a^ Maneval’s staining showed that all strains were encapsulated. The KOH string test [42] was used to verify the Gram reactions of strains that showed ambiguous Gram stain results. Photomicrographs of all strains are shown in Figure 2 and Figure 3.

### 3.2. Phylogeny

Phylogenetic analyses showed the six strains of Lake Vanda bacteria belong to four distinct phylogenetic lineages. BLAST searches indicate strain BYV50 is a species of the genus *Rhodococcus*, strain VP55 is of the family *Bradyrhizobiaceae* and aligns most closely with the genus *Rhodopseudomonas*, strain VY55 is a species of *Sphingobium*, and strains VW50, VS55, and VW55 are species of the genus *Brevundimonas*. 

Strain BYV50 showed 100% 16S rRNA gene sequence identity to Arctic seawater bacterium R7851 and 99.9% identity to three other *Rhodococcus* species, all of Arctic or Antarctic origin (Figure 2B). Strains VW55, VW50, and VS55 showed 100% 16S rRNA gene sequence identity to each other and to several additional *Brevundimonas* strains, including Mediterranean Sea bacterium *Brevundimonas mediterranea* str. V4.BO.18 (Figure 3B). In the same analysis, strain VP55 showed 99.4% identity to legume-associated *Rhodopseudomonas* sp. ORS 1416ri and *Rhodopseudomonas* sp. R-45977. Strain VY55 had an identical 16S rRNA gene sequence to Antarctic *Sphingobium* spp. FO10 and a 99.3% identity to Antarctic isolate *Sphingomonas* sp. Ant17 (Figure 3B).

### 3.3. Physiology

All Lake Vanda strains grew aerobically on medium R2A. With the exception of strain VP55, which did not grow on any sugar or alcohol tested, all strains were capable of using a range of carbon sources to support growth, and yeast extract supported good growth of all strains (Table 2). Anaerobic growth was not observed in any cultures, either by fermentation of glucose or anaerobic respiration in anoxic medium R2A supplemented with commonly used alternative electron acceptors, including dimethyl sulfoxide, nitrate, or fumarate (10 mM each). Moreover, sulfur reduction was not observed in tubes of SIM medium, which contains both ferrous ammonium sulfate and sodium thiosulfate.

**Table 2:** Carbon source utilization by Lake Vanda bacteria isolated in this study ^a^.

Carbon Source	*Rhodococcus* sp. BYV50	*Brevundimonas* spp. VW50, VW55, VS55	*Bradyrhizobiaceae* sp. VP55	*Sphingobium* sp. VY55
SUGARS				
Glucose	+	+	−	(+)
Fructose	++	(+)	−	−
Ribose	+	(+)	−	−
Galactose	+	+	−	(+)
Sucrose	++	−	−	(+)
Maltose	+	+	−	(+)
Lactose	+	(+)	−	(+)
Xylose	++	−	−	−
Mannose	+	(+)	−	−
ALCOHOLS				
Ethanol	+	+	−	(+)
Propanol	++	(+)	−	−
FATTY AND ORGANIC ACIDS				
Acetate	+	+	−	(+)
Pyruvate	+	+	+	(+)
Propionate	++	−	−	−
Butyrate	++	+	(+)	(+)
Lactate	++	+	+	−
Fumarate	+	+	−	−
Succinate	++	+	(+)	(+)
Benzoate	+	−	−	−
Yeast Extract	++	++	+	+

^a^ Growth was assessed as the difference of the time zero absorbance (OD_540_) from the final absorbance using the following scale: ++, ∆ OD_540_ > 0.15; +, ∆ OD_540_ 0.051–0.150; (+), ∆ OD_540_ 0.021–0.050; −, ∆ OD_540_ 0–0.02. Carbon sources had the following final concentrations: yeast extract, 0.1%; benzoate, lactose, 3 mM; maltose, sucrose, fructose, glucose, galactose, butyrate, succinate, 5 mM; all others, 10 mM. No strains grew with malate or citrate (10 mM each) as sole carbon source, and only strain BYV50 was capable of degrading urea. All strains tested negative for tryptophanase, gelatinase, cysteine desulfurase, and starch hydrolysis.

**Figure 4:**
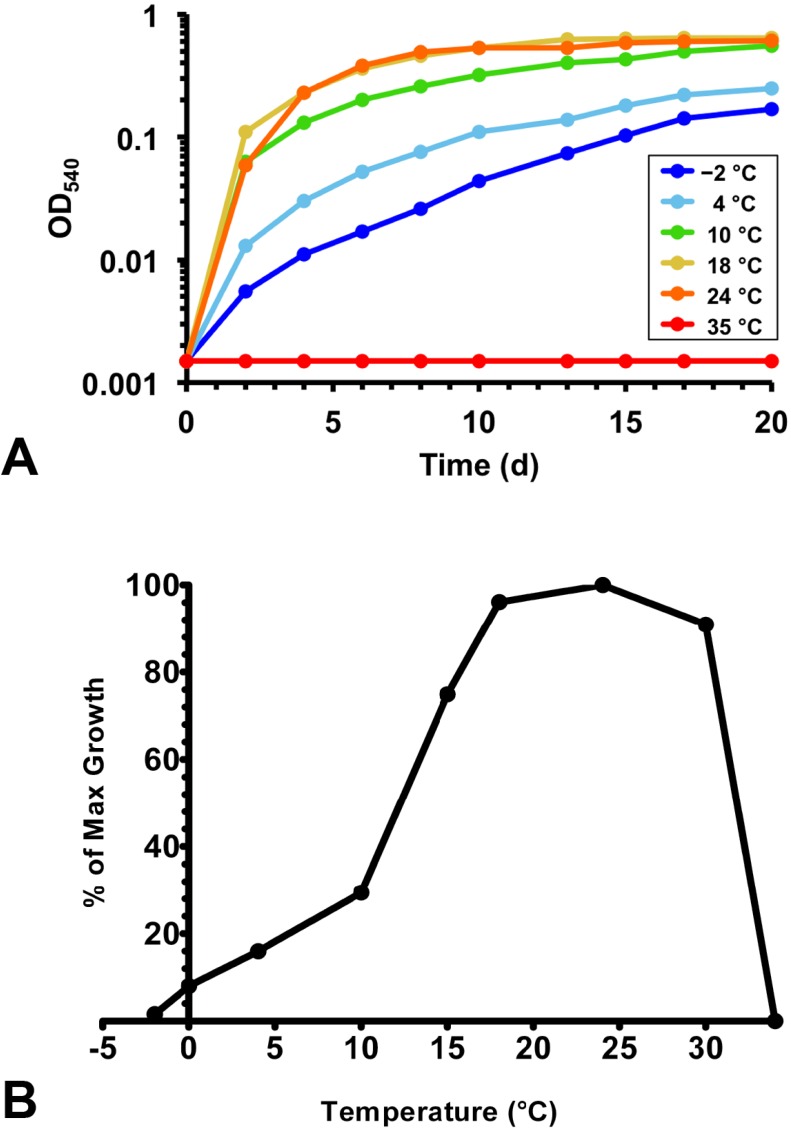
Growth response to temperature for Lake Vanda strain BYV50. (**A**) Growth of strain BYV50 over a range of temperatures in medium R2A containing 0.5% NaCl. Slow growth at −2 °C occurred, while no growth was observed at 35 °C; (**B**) Expression of growth rate data as percent maximum growth. Optical densities for triplicate cultures at each temperature were averaged following growth for seven days. The highest density was assigned a value of 100, and the percentage of maximum growth was calculated for each remaining temperature.

All Lake Vanda strains grew at the temperatures of their habitat. The *Brevundimonas* strains and *Rhodococcus* str. BYV50 grew within a broad temperature range and were capable of slow growth at −2 °C (Table 1 and Figure 4). The other Lake Vanda strains showed a lesser degree of cold adaptation; growth at 0 °C was very slow for strain VY55 and did not occur for strain VP55. Whereas all strains showed cold adaptation to varying degrees, optimal growth of all strains occurred near 20 °C (Table 1). *Rhodococcus* str. BYV50 had a fairly wide optimal temperature range, as robust growth occurred from 18 to 24 °C (Figure 4). The *Brevundimonas* strains had a maximum growth temperature of 35 °C, and therefore these strains showed the widest growth temperature range. Strains BYV50 and VY55 were capable of growth up to 32 °C and 30 °C, respectively, and strain VP55 grew within the narrowest temperature range of 2–29 °C.

The Lake Vanda strains showed a wide range of salinity tolerances. While none of the Lake Vanda isolates had a NaCl requirement, the addition of 0.5% NaCl (final) to culture media stimulated growth of all strains, and several strains showed at least a moderate degree of halotolerance. Strain BYV50 was strongly halotolerant and could grow in media containing up to 10% NaCl. The *Brevundimonas* and *Sphingobium* strains were less halotolerant and grew in media containing up to 6% NaCl. By contrast, strain VP55 was quite salt sensitive and failed to grow in media containing more than 1% NaCl.

## 4. Discussion

Our phylogenetic analyses showed the Lake Vanda strains to be species of *Nocardiaceae*, *Caulobacteraceae*, *Sphingomonadaceae*, and *Bradyrhizobiaceae*; these bacterial families contain primarily aquatic or terrestrial bacteria. It is therefore of interest to consider the origin of these isolates since they are removed from the “external environment” by nearly four meters of permanent ice cover.

Most of the Lake Vanda strains showed a close phylogenetic relationship to bacteria obtained from diverse Antarctic and non-Antarctic ecosystems. For example, *Rhodococcus* sp. BYV50 showed 100% 16S rRNA gene sequence identity to Arctic seawater bacterium R7851 isolated from the Western Greenland Sea [43]. Like the plant pathogen *Rhodococcus fascians*, strains BYV50 and R7851 are Gram positive and display yellow-to-orange pigmentation [43]. Other *Rhodococcus* species related to *Rhodococcus* sp. BYV50 were isolated from Antarctica, including *R. cercidiphylli* from an ice core obtained from central Dronning Maud Land [44] and Antarctic seawater bacterium R7241 from sites above the Gunnerus and Astrid Ridges [43]. The pervasive polar distribution of these closely related *Rhodococcus* strains suggests they may be important contributors to organic carbon degradation in cold, oligotrophic waters.

Phylogenetic analyses of the *Brevundimonas* strains showed 100% identity to each other and to several other *Brevundimonas* strains, including *B. mediterranea* str. V4.BO.18 and *Brevundimonas* sp. cp03. The latter strains were isolated from the northwestern basin of the Mediterranean Sea [45] and a magnetite mine drainage sample near an iron mine in the Hebei Province of China [46], respectively. It therefore appears that this versatile *Brevundimonas* species, which includes strains ranging from Antarctic lakes to the Mediterranean Sea, has a widespread and diverse aquatic distribution.

Lake Vanda strain VP55 formed translucent, pink colonies consisting of nonmotile, short rods that often had a tapered appearance. This isolate showed 99.4% identity to both *Rhodopseudomonas* sp. ORS 1416ri, isolated from legume root nodules in Tunisia [47], and *Rhodopseudomonas* sp. R-45977, rhizobia isolated from legumes in Belgium [48]. This connection is peculiar since the permanently cold Dry Valleys lakes support solely microbial life and would therefore not be expected to harbor strains so closely related to bacteria that develop mutualistic associations with plants. More in line with the ecophysiology of strain VP55, BLAST searches also revealed a close phylogenetic relationship of this organism to *Rhodopseudomonas* sp. 194, a bacterium isolated from an ice core. A formal description of strain 194 has not been published, and thus its physiology and exact habitat are unclear.

Despite its phylogenetic association with species of the purple bacterium *Rhodopseudomonas*, such as *R. palustris*, strain VP55 did not show the pigmented photoheterotrophic growth typical of purple nonsulfur bacteria [49] when incubated for six weeks in the light in an anoxic medium containing acetate, pyruvate, and succinate. Moreover, a spectral analysis of intact cells showed absorbance maxima within the range typical of carotenoids (400–550 nm) but no evidence for bacteriochlorophyll *a* (data not shown). Therefore, strain VP55 is presumably nonphototrophic. It is also of interest that strain VP55 was significantly restricted in its use of organic compounds. Unlike the other five Lake Vanda strains and unlike what is typical of purple nonsulfur bacteria [49], strain VP55 was unable to catabolize sugars, alcohols, or fatty acids and used only a few organic acids as carbon and energy sources. The phylogenetic position of strain VP55 leaves open the remote possibility that besides heterotrophy, the organism may also be able to grow chemolithotrophically, for example, on H_2_.

The bright-yellow-pigmented strain VY55 was phylogenetically identical to *Sphingobium* sp. FO10, isolated from the water column of Lake Fryxell, Antarctica, and showed 99.3% sequence identity to *Sphingomonas* sp. Ant17, a hydrocarbon-degrading bacterium isolated from soil near Scott Base, Antarctica; hence, these are possible sources of this organism in Lake Vanda. Interestingly, however, while some relatives of psychrotolerant strain VY55 are also adapted to cold temperatures, others are mesophilic. For example, *Sphingobium rhizovicinum* str. CC-FH12-1, isolated from rhizosphere soils of the fruit-bearing tree *Fortunella hindsii*, has a growth range of 22–37 °C [50]. Another relative of strain VY55, *Sphingomonas* sp. BP-7, was isolated from Seto Inland Sea water offshore of Matsuyama, Japan [51]. In support of the phylogenetic connection between strain VY55 and the marine strain BP-7, growth of strain VY55 occurred in media containing up to 6% NaCl, and colonies of both strains exhibited yellow pigmentation.

## 5. Conclusions

Our research shows that although most of the Lake Vanda bacteria isolated in this study are capable of growth at subzero temperatures, optimal growth of these strains occurred from 18 to 24 °C, a phenomenon that has been repeatedly observed in phylogenetically diverse bacteria isolated from other Dry Valleys lakes [16,17,18,19]. Since psychrophiles have optimal growth temperatures at or below 15 °C [52], all six Lake Vanda strains would best be described as psychrotolerant rather than psychrophilic. In addition to some measure of cold adaptation, the Lake Vanda strains demonstrated varying degrees of halotolerance, with one strain, *Rhodococcus* strain BYV50, capable of growth in media containing 10% NaCl. The broad range of halotolerance in these isolates may be a reflection of the pronounced salinity gradient within the Lake Vanda water column itself, which increases from freshwater to approximately 11% with depth (Figure 1).

Although the Lake Vanda strains described here are not optimally adapted to the 10–12 °C temperatures that occur at the depths from which they were isolated, they are nevertheless capable of growth at such temperatures and likely compete well under such conditions. The impressive versatility of these strains, characterized by their ability to grow (1) in temperatures ranging from freezing to comparatively warm, (2) in the presence or complete absence of NaCl, and (3) at supersaturated levels of O_2_, suggests that these isolates are well suited to the highly stratified and varied physiochemical conditions of the Lake Vanda water column. As a final note, with the exception of the metabolically limited strain VP55, all strains showed the capacity to catabolize a wide range of carbon sources, and therefore these nutritionally versatile bacteria are likely to be important consumers in the highly oligotrophic and oxic upper waters of this unusual Dry Valley lake.
